# Otorhinolaryngological and esophageal manifestations of epidermolysis bullosa

**DOI:** 10.1016/S1808-8694(15)31373-2

**Published:** 2015-10-17

**Authors:** Rodrigo Santana Fantauzzi, Mariana Oliveira Maia, Flávia Coelho Cunha, Rodrigo Vidal Simões, Denise Utsch Gonçalves, Amélio Ferreira Maia

**Affiliations:** 1 Otorhinolaryngologist, Member of the Clinical Team at Hospital Municipal de Contagem; 2 MD, Resident at the ENT Service at Hospital das Clínicas da UFMG; 3 Otorhinolaryngologist, Member of the Clinical Team at Hospital Júlia Kubitschek; 4 Otorhinolaryngologist, Member of the Clinical Team at Hospital Militar do Estado de Minas Gerais; 5 PhD, Adjunct Professor of the Department of Ophthalmology, Otorhinolaryngology, and Speech and Hearing Therapy at the UFMG Medical School, Otorhinolaryngologist and Full Advisor at the Graduate Program on Infectology and Tropical Medicine at the UFMG Medical School; 6 Otorhinolaryngologist, Head and Neck Surgeon, Coordinator of the Otorhinolaryngology and Head and Neck Surgery Clinic at Hospital Felício Rocho. Hospital Felício Rocho

**Keywords:** epidermolysis bullosa, esophagus, manifestations, otorhinolaryngology, treatment

## Abstract

Epidermolysis bullosa (EB) is a group of skin diseases with different clinical manifestations and varied inheritance patterns. Blisters may appear spontaneously or following minimal trauma to the skin or mucosa.

**Aim:**

this paper aims to describe the otorhinolaryngological manifestations and esophageal complications related to EB, and the experience in treating patients with esophageal stenosis secondary to this disease.

**Materials and method:**

this descriptive study enrolled 60 patients with EB seen from June 1999 to December 2006 at the Head and Neck Surgery Service of X Hospital, a reference center for EB.

**Results:**

the patients’ mean age was 14.5 years. Twenty-eight (46.6%) were females and 32 (53.4%) were males. Eight (13.4%) were diagnosed with epidermolysis bullosa simplex, while 51 (85%) had epidermolysis bullosa dystrophica; one (1.6%) patient had one acquired EB. Lips, mouth, tongue and ears were the most frequently involved sites (32 patients - 53.3%). Dysphagia was found in 28 patients (46.6%). After esophageal dilatation the symptoms subsided.

**Conclusion:**

EB is a rare disease and patients must be sent for treatment at reference centers. Physicians treating patients for EB must be aware of the measures required to improve the quality of the treatment provided without putting the patients in harm’s way.

## INTRODUCTION

Epidermolysis bullosa (EB) is a group of skin diseases with different clinical manifestations and varied inheritance patterns^1^, ^2^, ^3^, ^4^. Blisters may appear spontaneously or following minimal trauma to the skin or mucosa^4^, ^5^. EB affects one in every 50,000 livebirths^4^. Blisters appear secondary to the formation of cleavage planes in skin and mucosa^2^, ^3^, ^5^, ^6^, ^7^. EB can be divided into three large clinical groups: EB simplex (dominant autosomal) with blisters located in the epidermis that do not leave scars; junctional EB (recessive autosomal) in which blisters form in the lamina lucida; and EB dystrophica (dominant or recessive autosomal) characterized by the presence of atrophy, milium cysts, ungueal dystrophies, pigmentary alterations and mucosal injuries^2^, ^4^.

Twenty-three phenotypes of EB are known, with varied clinical manifestations ranging from mild to lethal cases^4^. Diagnosis is done based on lesion clinical findings and pathology tests^4^. Electronic microscopy is the golden standard for diagnosis, but immunohistochemistry (monoclonal antibodies specifically) and molecular biology have provided sophisticated definitions of normal skin dermo-epidermal junction structure and function and alterations found on each EB group and subgroup^4^.

Otorhinolaryngological manifestations are frequent in EB^6^, ^7^, ^8^, ^9^, ^10^, ^11^. Such manifestations are related to the appearance of mucosal blisters (mainly in the oropharynx and esophagus), followed by rupture and hypertrophic scarring, leading to frenulum shortening, microstomia, esophageal stenosis, laryngeal stenosis, and nasal vestibule stenosis. Esophageal stenosis is a complication usually found in advanced cases, and remains as one of the main challenges in treating this condition^12^. Descriptive studies analyzing this infirmity have not yet been published in Brazil.

This paper aims to describe the otorhinolaryngological manifestations of epidermolysis bullosa, defining the best clinical and/or surgical approaches to consequently reduce patient morbidity and mortality.

## MATERIALS AND METHOD

This is a descriptive study that enrolled 60 EB patients followed and treated from June 1999 to December 2006. The project was approved by the Ethics Committee of our institution under permit 136/05.

All patients were referred to the study by the Minas Gerais Association of Families, Friends, and Carriers of Epidermolysis Bullosa (AMPAPEB).

The 60 patients underwent a thorough otorhinolaryngological examination. Videofluoroscopy and contrast-enhanced x-rays of the esophagus, stomach, and duodenum were taken.

Esophageal dilatation was required in selected cases. Patients referred to the procedure had clinical and radiologic evidences of severe esophageal stenosis. Esophageal dilatation was performed using olives. All procedures were conducted with general anesthesia with propofol, succinylcholine, and midazolam. The procedures were carried out under apnea and mask ventilation until the patients recovered. After the procedure patients were prescribed steroids between 0.5 and 1mg/kg/5 days, analgesic drugs (paracetamol or dipyrone) and ranitidine (150mg/10ml) or omeprazole (20mg/12/12hs) for 30 days. Treatment course was prolonged depending on the presence of dyspeptic symptoms. Patients experiencing choking and dyspeptic symptoms without significant dysphagia took proton pump blockers or H2 receptor blockers in the previously prescribed dosages.

## RESULTS

Twenty-eight (46.6%) of the 60 patients enrolled in the study were females, whereas 32 (53.4%) were males. Median age was 14.5 years and time of symptom onset varied between birth and 22 years of age.

Eight (13.4%) were clinically diagnosed with EB simplex, 51 (85%) with recessive EB dystrophica and one (1.6%) with acquired EB.

**Figure 1:**
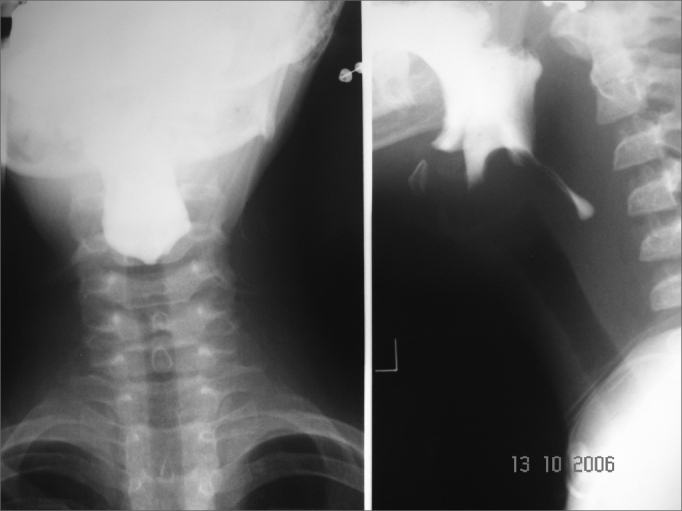
Esophageal stenosis in the cricoid constriction

Esophageal manifestations were found in 28 (46.6%) patients. All patients underwent esophageal dilatation after esophageal stenosis was confirmed through x-ray images (Fig. 1).

**Figure 2:**
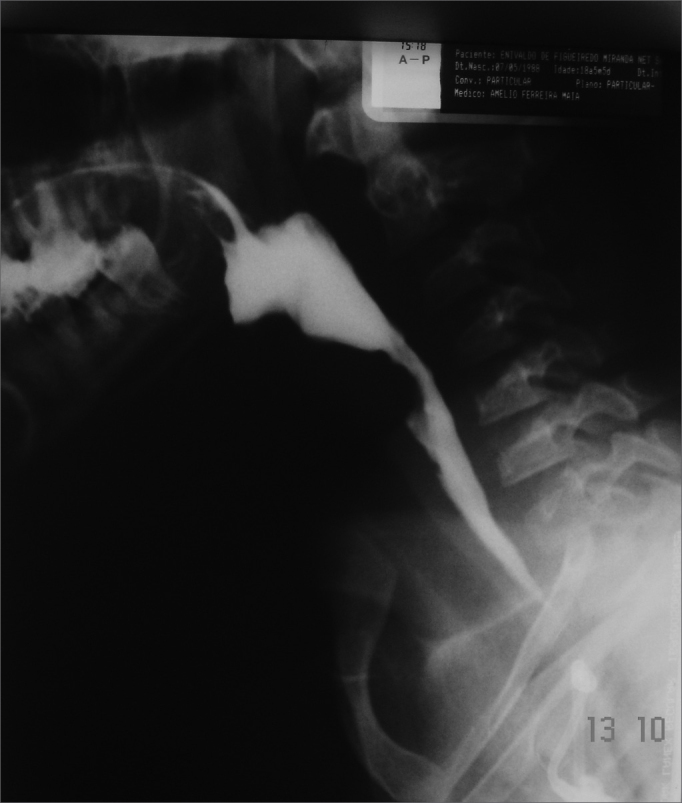
Patient in the previous figure after dilatation with metal olives

Two esophageal complications were observed: one patient submitted to dilatation evolved to an esophageal blister post-operatively and had to be hospitalized and clinically treated until the blister burst and the patient was able to swallow. Another patient had an esophageal perforation that evolved to pneumomediastinum and pneumoperitonium from the carina to the transverse mesocolon. This patient complained of intense retrosternal and chest pain while performing respiratory movements post-operatively. Chest x-rays were done to no diagnostic avail. A chest CT scan done subsequently showed a perforation. After starting treatment with clindamycin and gentamicin, the patient was referred to a drainage procedure covering the chest, mediastinum, and abdomen (retroperitonium). The patient evolved well and was discharged 30 days later. Dysphagia status was improved for all patients submitted to esophageal dilatation (Fig. 2).

**Figure 3:**
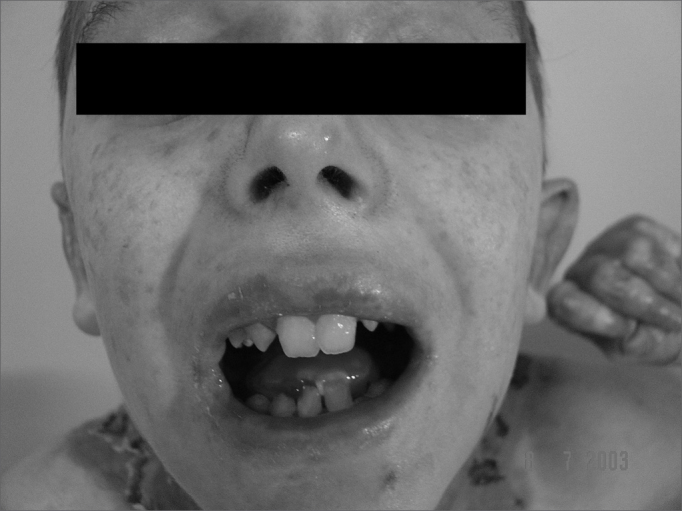
Microstomia, shortened frenulum, perioral and oral mucosal ulcerations

**Figure 4:**
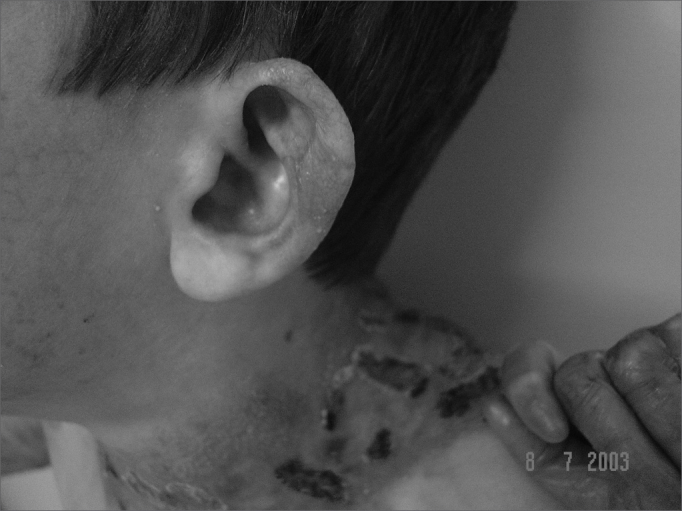
Scar lesion in the ear (milium).

Lips, mouth, tongue, and ear were the most frequently involved sites (Fig. 3 and 4), with blisters found in 32 (53.3%) patients. Blisters were found in the external ear canal of two (3.3%) patients; none of them had signs of stenosis in the ear canal. Ulcerative lesions and nostril blisters were encountered in 11 (18.3%) patients, three (27.2%) of which evolved to nostril stenosis. One (1.6%) patient had laryngeal-tracheal alterations (anterior commissure stenosis) (Table 1).

**Table 1:** Distribution of 60 patients with epidermolysis bullosa in relation to gender, subtype, and lesion site.

	Clinical characteristics	Patients
		60(100%)
Gender	Female	28 (46.6%)
	Male	32 (53.4%)
Subtype	Epidermolysis bullosa simplex	08
	Epidermolysis bullosa dystrophica	51
	Acquired Epidermolysis bullosa	01
Lesion sites	Esophagus	28
	Lips	32
	External ear canal	02
	Nostrils	11
	Larynx	01

No complications related to anesthesia were observed, and none of the patients developed skin lesions after the procedures.

## DISCUSSION

Epidermolysis bullosa is observed in a heterogeneous set of systemic diseases characterized by abnormal skin and mucosal frailty^3^. Research has recently shed light on important genetic defects^4^. Mutations on collagen type VII have been seen in cases of recessive and dominant EB dystrophica, while EB simplex has been associated with mutations on genes for keratin 5 or 143. Investigation has shown that some forms of junctional EB are caused by mutations in the laminin gene^3^. These findings have practical implications in the development of accurate pre-natal diagnosis and gene therapy^3^.

Depending on the type of epidermolysis bullosa, patients may develop phlyctenula in their mouths, esophagus, nostrils, ears, and larynx, resulting in significant morbidity^6^, ^7^, ^8^, ^9^, ^10^, ^11^, ^13^, ^14^, ^15^. Iron deficiency anemia is common with the blood losses subsequent to blister rupture^7^.

In relation to extracutaneous involvement, the upper gastrointestinal tract is the most frequently involved site^7^, ^10^. Phlyctenula and microstomia may occur and lead to dysphagia and odynofagia^7^. Radiologic findings include stenosis, blisters, uneven mucosal surface, and hiatal hernia. The esophageal mucosa may be extremely fragile in patients with EB dystrophica, and perforation has been reported after endoscopy^10^.

Esophageal blisters usually evolve to scar tissue and stenosis and may secondarily lead to dysphagia, one of the biggest problems in patients with recessive EB dystrophica7. Complications such as esophageal perforation, pneumomediastinum, pneumoperitonium, and mediastinitis must be diagnosed as early as possible, as they may be fatal conditions^12^.

Teeth are often compromised in EB patients^7^, ^14^, ^16^. Preventive care is needed and frequent visits to a dental care provider are required to keep the teeth clean. Early fluorine application is indicated for pediatric patients^7^, ^14^, ^16^. All patients included in our study had poor dental status. EB patients have a hard time brushing their teeth, as even minimal trauma results in lesions.

Laryngeal involvement is rare. It occurs more frequently in junctional EB than on EB dystrophica or EB simplex^8^, ^9^, ^13^, ^17^. Acute laryngeal involvement by blisters evolving to hoarseness and stridor requires immediate otorhinolaryngological intervention and possibly a tracheostomy^2^.

EB patients may be offered regular anesthesia, however with greater risk for complication^17^, ^18^. A recent review looked at 129 anesthesia administrations in 32 EB patients and found no complications in patients using orotracheal intubation (10 cases), face mask, nerve blockage, intramuscular or intravenous agents, and local anesthesia. Similarly, no intubation-related complications were found in another report including 113 cases of oral intubation and 18 of nasal intubation done on 33 EB patients^17^. It is possible that the pseudostratified columnar epithelium that covers most of the larynx and trachea reduces the formation of friction blisters^17^. Despite their low risk of complication, any instrument that contacts skin and mucosa (face mask, laryngoscope, and endotracheal tubes) must be well lubricated^17^. Lubricated gauze has been indicated to fixate electrodes in cardiac monitoring, pressure cuff and venous accesses^17^.

Complications such as esophageal perforation, pneumomediastinum, pneumoperitonium, and mediastinitis must be diagnosed early, as they may be fatal^12^.

## CONCLUSION

EB is a rare disease and patients must be sent to reference centers for treatment. Therefore, it is fundamental that the physicians involved in providing care to these patients are aware of the necessary measures to optimize the treatment without further harming the patients.
